# Magnetometer Calibration for Small Unmanned Aerial Vehicles Using Cooperative Flight Data†

**DOI:** 10.3390/s20020538

**Published:** 2020-01-18

**Authors:** Roberto Opromolla

**Affiliations:** Department of Industrial Engineering, University of Naples Federico II, Piazzale Tecchio 80, 80125 Naples, Italy; roberto.opromolla@unina.it; Tel.: +39-0817683365

**Keywords:** unmanned aerial vehicles, magnetometers, magnetic bias, magnetic heading, multi-UAV cooperation, vision-based relative sensing, GNSS-based relative sensing, Levenberg-Marquardt

## Abstract

This paper presents a new method to improve the accuracy in the heading angle estimate provided by low-cost magnetometers on board of small Unmanned Aerial Vehicles (UAVs). This task can be achieved by estimating the systematic error produced by the magnetic fields generated by onboard electric equipment. To this aim, calibration data must be collected in flight when, for instance, the level of thrust provided by the electric engines (and, consequently, the associated magnetic disturbance) is the same as the one occurring during nominal flight operations. The UAV whose magnetometers need to be calibrated (chief) must be able to detect and track a cooperative vehicle (deputy) using a visual camera, while flying under nominal GNSS coverage to enable relative positioning. The magnetic biases’ determination problem can be formulated as a system of non-linear equations by exploiting the acquired visual and GNSS data. The calibration can be carried out either off-line, using the data collected in flight (as done in this paper), or directly on board, i.e., in real time. Clearly, in the latter case, the two UAVs should rely on a communication link to exchange navigation data. Performance assessment is carried out by conducting multiple experimental flight tests.

## 1. Introduction

The use of unmanned aerial vehicles (UAVs) has been increasing exponentially in recent years, and thus they are having a strong economic impact on the global market [1]. Indeed, they can be used for a large variety of military and civilian applications [2], such as border patrol [3], search and rescue [4], infrastructure monitoring [5], package delivery [6] and precision agriculture [7]. To fully enable the abovementioned mission scenarios, it is widely agreed that strong research efforts must be dedicated to significantly enhance the level of autonomy of UAVs regarding their guidance, navigation and control functions [8], on the one hand, and on the other hand, to the definition of a framework where they can be safely integrated in the civil airspace (see for example the NASA Unmanned Traffic Management program [9] and Corus project by SESAR [10]).

In this respect, this work lies in the framework of research activities, carried out at the Department of Industrial Engineering of the University of Naples “Federico II”, aiming at investigating the possibility to exploit cooperative strategies to improve navigation performance of mini/micro UAVs (MAVs) [11,12]. If these vehicles fly under nominal Global Navigation Satellite System (GNSS) coverage, autonomous and safe navigation is enabled by integrating measurements from low-cost GNSS receivers and commercial-grade micro-electro-mechanical systems (MEMS)-based inertial (i.e., gyroscopes and accelerometers) and magnetic sensors, within Extended Kalman Filters [13,14] or, more recently, Particle Filters [15], and using either loosely- or tightly-coupled architectures [16]. In this respect, since onboard gyros do not have enough sensitivity to measure the Earth rate vector, magnetometers play a key role, despite being typically characterized by low bandwidth and high measurement noise. Indeed, their capability to measure the Earth magnetic field, and, consequently to identify the North direction, can be used to bound the heading error by including a magnetic heading estimate among the measurements used by the onboard navigation system of the MAV to get its navigation state. Unfortunately, the presence of magnetic sources, either external or placed on board the MAV, can significantly affect the measurements of a three-axis magnetometer. Together with intrinsic sensor error sources (e.g., biases, scale factor deviations, misalignment and non-orthogonality of the sensor axes) for which a proper ground calibration is always required, these magnetic sources generate disturbances which lead to additional systematic errors in the measurements of the components of the Earth magnetic field in the Body Reference Frame (BRF) of the MAV. Consequently, the measured magnetic heading will be referred to an apparent magnetic North direction, thus leading to an angular heading error which may be up to 10°. For these reasons, such standalone navigation solutions can provide limited accuracy levels, i.e., 5–10 m and 1°–5° for MAVs position and attitude, respectively [17]. Although, these accuracy levels enable real-time stabilization and control, they do not allow meeting the requirements imposed by several applications (like 3D mapping [18]), unless ad-hoc solutions are adopted. For instance, multi-antenna-based GNSS techniques allow reaching sub-degree level in the attitude accuracy [17], but they pose installation constraints which may not be met considering the allocation resources of many micro/small UAVs [19]. So, in the most general case, considering performance of low-cost IMUs on board MAV, the uncertainties in the roll and pitch estimates provided by the onboard autopilot are typically bounded at the degree-level [20] (due to the presence of gravity), while the error in the heading angle estimate can be up to several degrees [21].

In order to address this problem, this paper investigates the possibility to improve the accuracy in the estimate of the magnetic heading provided by magnetometers. The calibration of magnetic sensors is typically based on the assumption that the intrinsic error sources can be characterized using an ellipsoidal error model [22,23]. Consequently, the parameters of the model ellipsoid can be computed by applying a non-linear optimization process, which requires in input a set of measurements taken with different pointing conditions of the sensor’s axes in order to properly sample the ellipsoid surface. However, these calibration procedures cannot cope with systematic errors induced by onboard magnetic sources which act during nominal flight operations of the MAV [24,25]. A closed-form analytical solution for the calibration problem of magnetometers on board UAV, conceived to potentially account also for the contribution of the electric motors, can be found in [26]. However, this method has not been tested in flight, and no information is provided by the authors regarding the achievable magnetic heading accuracy.

For this reason, an original calibration approach to estimate magnetic biases caused by onboard disturbances is proposed in this paper. This method exploits cooperation between two MAVs, and it requires calibration data to be collected in flight executing a predefined coordinated maneuver. In the following, the vehicle whose magnetometers need to be calibrated is called “chief”, while the cooperative vehicle is called “deputy”. The chief must be able to detect and track the deputy using a monocular camera (and, consequently, proper vision-based algorithms [27,28]) to estimate the unit vector corresponding to the chief/deputy relative position (i.e., the so-called line-of-sight, LOS) in the camera reference frame. Also, during the calibration flight, both the vehicles must be kept under nominal GNSS coverage to allow estimating their relative position in local (North-East-Down, NED) coordinates. Using the chief/deputy relative position information in camera and NED coordinates, the calibration problem can be mathematically formulated as a system of non-linear equations as described in detail in Section 2. This problem is addressed using a customized implementation of the Levenberg-Marquardt (LM) method [29] (i.e., a state-of-the-art least-squares solver) based on the formulation proposed by Gavin [30]. It is worth outlining that if both the MAVs are equipped with a camera, this approach can be used to calibrate the magnetic sensors on board both the two vehicles, before being able to carry out their cooperative or independent mission. Clearly, although this method is conceived to exploit cooperation between two MAVs, the proposed concept is perfectly applicable even if the deputy is another type of autonomous vehicle (e.g., ground or marine) or even if it is a fixed ground station, provided that it can acquire GNSS information while being visually tracked. Finally, it is also important to underline that the calibration approach can be applied either off-line (as done in this paper), or directly on board, i.e., processing in real time the acquired flight data. Clearly, the applicability of this latter option requires that the two UAV are able to exchange information about their navigation status using a communication link. An experimental campaign of flight tests is carried out to assess the applicability and performance of the proposed cooperative calibration method. The flight tests are carried out using both fixed and flying deputies. Preliminary results of this work were initially presented in a conference paper [31].

The rest of the paper is organized as follows: Section 2 formulates the magnetic bias calibration problem, and it describes in detail the proposed cooperative strategy and the associated LM-based algorithmic solution. Section 3 presents the setup used for data collection and the adopted experimental strategy, and it discusses the related results. Finally, Section 4 provides a conclusion and indications about future works.

## 2. Magnetometers Calibration Method

### 2.1. Magnetic Heading Definition

The calibration problem addressed in this work relies on the assumption that magnetic sensor’s measurements are not affected by disturbances from the environment where the MAV is operating. Clearly, this condition is not met if the MAV must fly within areas characterized by large external magnetic sources (e.g., close to power plants, power-lines or other similar facilities). Under this assumption, the magnetic field measured by a three-axes magnetometer (*H*) can be expressed using Equation (1):

(1) $$\underline{H}=\left[\begin{array}{c} H_{x}\\ H_{y}\\ H_{z} \end{array}\right]={\underline{H}}_{E}+\underline{\Delta H}$$

where *H_x_*, *H_y_* and *H_z_* are its components in BRF, *H_E_* is the Earth’s magnetic field, and ∆*H* is the magnetic bias. ∆*H* is caused by residual intrinsic error sources and (mostly) by onboard magnetic disturbances, thus being constant in BRF.

If the attitude of an MAV (namely, the orientation of its BRF with respect to NED) is parametrized by a 321 sequence of Euler Angles, i.e., heading (*ψ*), pitch (*θ*) and roll (*φ*), the Body-Stabilized Reference Frame (BSRF) can be defined as the reference frame obtained by projecting the BRF axes in the North-East plane thanks to the pitch and roll angle estimates provided by the chief onboard autopilot. Thus, the projection of *H* in the BSRF (*H_s_*) can be computed using Equation (2):

(2) $${\underline{H}}_{s}=\left[\begin{array}{c} Hx_{s}\\ Hy_{s}\\ Hz_{s} \end{array}\right]={\left(\underline{\underline{M_{\varphi }}}\underline{\underline{M_{\theta }}}\right)}^{-1}\left[\begin{array}{c} H_{x}\\ H_{y}\\ H_{z} \end{array}\right]$$

where *M_θ_* and *M_φ_* (i.e., the elemental rotation matrixes corresponding to *θ* and *φ*, respectively) can be written as follows:

(3) $$\begin{array}{l} \underline{\underline{M_{\varphi }}}=\left[\begin{array}{ccc} 1 & 0 & 0\\ 0 & \cos\left(\varphi \right) & \sin\left(\varphi \right)\\ 0 & -\sin\left(\varphi \right) & \cos\left(\varphi \right) \end{array}\right]\\ \underline{\underline{M_{\theta }}}=\left[\begin{array}{ccc} \cos\left(\theta \right) & 0 & -\sin\left(\theta \right)\\ 0 & 1 & 0\\ \sin\left(\theta \right) & 0 & \cos\left(\theta \right) \end{array}\right] \end{array}$$

**Figure 1:**
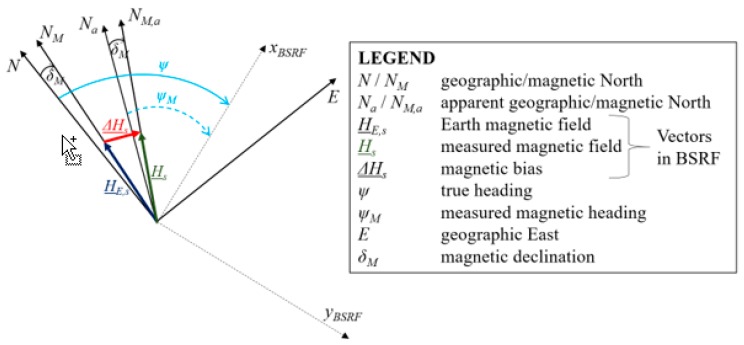
Effect of onboard magnetic sources on the magnetic heading estimation process. They cause the heading angle to be estimated with respect to an apparent geographical North rather than the true North direction. Please note that the figure is not in scale and the sizes of the vectors are chosen for the sake of clarity.

Clearly, the in-plane components of *H_s_* (namely, *Hx_s_* and *Hy_s_*) can be used to compute the magnetic heading (*ψ_M_*), i.e., the heading angle estimate obtained using the measurements of a three-axes magnetometer, as shown by Equation (4)

(4) $$\psi_{M}=\tan^{-1}\left(-\frac{Hy_{s}}{Hx_{s}}\right)+\delta_{M}$$

where *δ_M_* is the local magnetic declination, i.e., the angle (on the local horizontal plane) between the magnetic North (*N_M_*) and the true (geographic) North (*N*). Unfortunately, due to the presence of *∆H*, such heading estimate differs from the true heading (*ψ*) as it is referred to an apparent geographic North direction (*N_a_*) rather than to the true one, as depicted by Figure 1. By substituting Equation (1) in Equation (4), *ψ_M_* can be expressed using Equation (5):

(5) $$\psi_{M}=\tan^{-1}\left(\frac{-\left(H_{E,ys}+\Delta Hy_{S}\right)}{\left(H_{E,xs}+\Delta Hx_{S}\right)}\right)+\delta_{M}$$

where *H_E,xs_* (Δ*Hx_S_*) and *H_E,ys_* (Δ*Hy_S_*) are the in-plane components of *H_E_* (*ΔH*) in BSRF. Consequently, the proposed calibration approach aims at increasing the magnetic heading accuracy by computing the in-plane components of the magnetic bias expressed in BSRF (Δ*H**_s_*). Indeed, this allows obtaining a calibrated magnetic heading (*ψ_M,c_*) as shown by Equation (6):

(6) $$\psi_{M,c}=\tan^{-1}\left(\frac{-Hy_{S}+\Delta Hy_{S}}{Hx_{S}-\Delta Hx_{S}}\right)+\delta_{M}$$

Figure 1 provides a simplified representation of the problem addressed in this paper.

### 2.2. Cooperative Calibration Strategy

The determination of Δ*Hx_S_* and Δ*Hy_S_* must rely on data collected in flight, i.e., when all the chief onboard systems operate nominally, thus ensuring that the level of the associated magnetic disturbances is also nominal. To carry out this task, a cooperative calibration strategy is here proposed. Specifically, the two vehicles must execute a coordinated maneuver, during which the visual camera mounted on board the chief is used to detect and track the deputy through a sequence of frames. Two conditions must be strictly met regarding the definition of the flight path for the two vehicles:

- They must fly under nominal GNSS coverage.
- The deputy must stay within the camera Field-of-View (FOV).

Besides these mandatory points, some additional guidelines can be followed regarding the flight path definition to improve accuracy in the result of the calibration process, as better explained in Section 3.

For each frame acquired by the camera on board the chief, the position of the deputy projection on the image plane can be detected. To this aim, if the magnetic calibration is done in real time, an autonomous visual detection and tracking algorithm must be implemented on board. Due to the cooperative nature of the proposed calibration strategy, this image processing task can be carried out exploiting the knowledge of the deputy (e.g., in terms of size, shape and color) as well as its possibility to exchange navigation data with the chief by means of a communication link, as proposed in [27] and [28]. Instead, if the calibration is carried out off-line (i.e., after flight data acquisition), the visual detection task can be realized using a supervised approach (i.e., the position of the deputy projection on the image plane is extracted manually by a human operator). In both the cases, the image position information can be used to compute the unit vector (*u_CRF_*) representing the deputy LOS in the camera reference frame (CRF) using the intrinsic camera calibration parameters [32]. If autonomous visual algorithms are used, the angular accuracy in the LOS estimate can be of the order of the camera instantaneous FOV [28], while a supervised (off-line) approach can even lead to better accuracy due to the possibility to identify the deputy position at sub-pixel level.

Since the two vehicles are both flying under nominal GNSS coverage, the relative position vector and, consequently, the unit vector representing the deputy LOS in NED with respect to the chief (*u_NED_*) can also be determined. In this respect, it is worth outlining that the level of accuracy characterizing the estimates of *u_NED_* depends on the adopted relative positioning method. Specifically, the chief-deputy relative position vector can be obtained either computing the difference between the two position fix or applying Differential Global Positioning System (DGPS) [33], or Carrier-Phase DGPS (CDGPS) [34] algorithms. Clearly, in the latter case, GNSS raw data, namely pseudo-range and carrier-phase measurements, must be available. With regards to the guidelines for flight path definition mentioned earlier, the angular accuracy in *u_NED_* can be also improved by keeping the deputy as far as possible from the chief. Of course, the maximum allowable distance is determined by the size of the deputy, the camera resolution and the local background (which can be more or less cluttered) in the image plane.

At this point, if the visual and positioning data are properly synchronized (meaning that a GNSS-based relative position measurement is associated to each visual frame), the relationship between *u_CRF_* and *u_NED_* can be written as follows:

(7) $${\underline{u}}_{CRF}={\underline{\underline{M}}}_{M}{\underline{\underline{M}}}_{\psi \theta \varphi }{\underline{u}}_{NED}$$

where ${\underline{\underline{M}}}_{M}$ is the camera-body extrinsic calibration matrix and ${\underline{\underline{M}}}_{\psi \theta \varphi }$ is the UAV attitude matrix (i.e., the full rotation matrix corresponding to the 321 sequence of Euler angles defined in Section 2.1). If the camera is mounted according to a strapdown configuration, ${\underline{\underline{M}}}_{M}$ (representing the orientation of the CRF with respect to the BRF) is constant and it can be computed by carrying out an ad-hoc extrinsic calibration procedure either off-line [35] or on-line [36]. Using the pitch and roll angles available from the chief onboard navigation system, ${\underline{\underline{M}}}_{\psi \theta \varphi }$ can be expressed as a function of the unknown calibrated magnetic heading as shown by Equation (8):

(8) $${\underline{\underline{M}}}_{\psi \theta \varphi }\left(\psi_{M,c}\right)={\underline{\underline{M}}}_{\varphi }{\underline{\underline{M}}}_{\theta }\left[\begin{array}{ccc} \cos\left(\psi_{M,c}\right) & \sin\left(\psi_{M,c}\right) & 0\\ -\sin\left(\psi_{M,c}\right) & \cos\left(\psi_{M,c}\right) & 0\\ 0 & 0 & 1 \end{array}\right]$$

Consequently, if Equations (6) and (8) are substituted within Equation (7) for *k* time instants at which synchronized visual and GNSS data have been acquired, the magnetic bias calibration process can be formulated as a system of 3*k* non-linear equations in the two unknowns, i.e., Δ*Hx_S_* and Δ*Hy_S_*. In fact, for each of the *k* time instants, a triplet of equations is obtained by setting to zero the residual vector function (*r*) as shown by Equation (9):

(9) $$\underline{r}=f\left(\Delta Hx_{S},\Delta Hy_{S}\right)={\underline{u}}_{CRF}-{\underline{\underline{M}}}_{M}{\underline{\underline{M}}}_{\psi \theta \varphi }{\underline{u}}_{NED}=0$$

**Figure 2:**
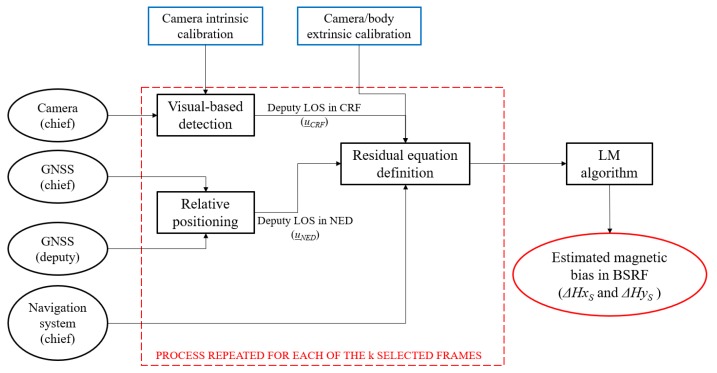
Cooperative strategy for magnetic sensor calibration. The camera and camera/body calibrations (blue rectangular boxes) are carried out offline. The final output is enclosed within an ellipse red box.

Hence, the estimation of the unknown magnetic bias components is entrusted to a customized implementation of the LM algorithm, which is an iterative technique to solve non-linear least squares problems. In the following, the symbol *r* is used to indicate directly the [3*k* × 1] residual vector obtained writing (9) for all the selected images. A block diagram which summarizes the proposed cooperative calibration approach is depicted in Figure 2.

### 2.3. LM-Based Solution

The LM algorithm allows formulating the least-squares minimization of the residual vector function in Equation (9) as a scalar problem by introducing an equivalent scalar cost function, namely the chi-squared error (*χ*^2^). This cost function is the sum of the weighted squares of the errors (also called residuals) between the measured data, i.e., the left-hand side in Equation (7) in this case, and the curve-fit function, i.e., the right-hand side in Equation (7) in this case, and it can be defined as follows:

(10) $$\chi^{2}\left(\Delta Hx_{S},\Delta Hy_{S}\right)={\underline{r}}^{T}\underline{\underline{W}}\underline{r}$$

where $\underline{\underline{W}}$ is a [3*k ×* 3*k*] diagonal weight matrix, whose elements are the reciprocal of the weights representing the level of confidence associated to each measured vector (*u_CRF_*). Here, $\underline{\underline{W}}$ is set to be an identity matrix, meaning that the same weight/importance is assigned to each visual estimate of the deputy LOS in CRF. This choice is in line with the fact that the accuracy in the estimate of the position of the deputy projection on the image plane does not vary significantly with factors like relative distance and local background (which can mostly influence the detection probability, if the image processing task is carried out on line, rather than its accuracy [27]).

In this work, the minimization of *χ*^2^ is carried out exploiting the numerical formulation of the LM technique proposed by [30]. According to this formulation, an updated estimate of the unknown in-plane components of Δ*H* in BSRF is obtained at each LM iteration using Equation (11),

(11) $$\left[\begin{array}{c} \Delta Hx_{S}{}^{j}\\ \Delta Hy_{S}{}^{j} \end{array}\right]=\left[\begin{array}{c} \Delta Hx_{S}{}^{j-1}\\ \Delta Hy_{S}{}^{j-1} \end{array}\right]+{\underline{h}}_{LM}$$

where *j* is the iteration counter, and *h_LM_*, i.e., the LM-based correction vector, is computed using the Marquardt’s relationship [29]:

(12) $${\underline{h}}_{LM}={\left({\underline{\underline{J}}}^{T}\underline{\underline{W}}\underline{\underline{J}}+\lambda D_{J^{T}WJ}\right)}^{-1}{\underline{\underline{J}}}^{T}\underline{\underline{W}}\underline{r}$$

where *λ* is the LM damping parameter, $\underline{\underline{J}}$ is the [3*k* × 2] Jacobian matrix defined as in Equation (13), and *D_JTWJ_* is a [2 × 2] matrix containing only the diagonal elements of $\underline{\underline{J^{T}WJ}}$:

(13) $$\underline{\underline{J}}=\left[\begin{array}{c} \frac{\partial {\underline{r}}^{1}}{\partial \Delta Hx_{S}}\\ \vdots \\ \frac{\partial {\underline{r}}^{k}}{\partial \Delta Hx_{S}} \end{array}\begin{array}{c} \frac{\partial {\underline{r}}^{1}}{\partial \Delta Hy_{S}}\\ \vdots \\ \frac{\partial {\underline{r}}^{k}}{\partial \Delta Hy_{S}} \end{array}\right]$$

Considering that the LM algorithm is a combination of two minimization techniques, namely the gradient descent method and the Gauss-Newton method, the value of the damping factor is a key parameter to determine its convergence. In fact, for large values of *λ*, the correction vector obtained from Equation (12) is closer to the one provided by the gradient descent method, meaning that *χ*^2^ is reduced in the direction opposite to its gradient. Instead, for small values of *λ*, the correction vector obtained from Equation (12) is closer to the one provided by the classical Gauss-Newton method.

Once the LM-based correction is computed, the updated solution provided by Equation (11) is not passed at the next step of the iterative process unless a specific condition on the associated variation on *χ*^2^ is met. The adopted confirmation criterion is given by the following relation [30]:

(14) $$\frac{\chi^{2}\left(\Delta Hx_{S}{}^{k-1},\Delta Hy_{S}{}^{k-1}\right)-\chi^{2}\left(\Delta Hx_{S}{}^{k},\Delta Hy_{S}{}^{k}\right)}{2{\underline{h}}_{LM}\left(\lambda {\underline{h}}_{LM}+{\underline{\underline{J}}}^{T}\underline{\underline{W}}\underline{r}\right)}>\varepsilon_{0}$$

where *ε_o_* is a positive threshold set by the user. For instance, this condition allows discarding an update which would cause an increase in *χ*^2^. Based on the result of Equation (14), the value of the damping factor is changed at each iteration to accelerate convergence. Specifically, if the condition in Equation (14) is met (not met), *λ* is decreased (increased) using the scaling factor *λ_DN_* (*λ_UN_*). Clearly, large scaling factors can provide convergence in fewer iterations, but the process may encounter numerical instabilities. 

With regards to the initialization of the iterative procedure, the initial values of Δ*Hx_S_* and Δ*Hy_S_* are set to zero, though a non-zero initial guess may be used if available, e.g., obtained by an on-ground calibration. The initial value of *λ* (*λ*_0_) is also freely selected by the user.

Finally, the iterative process is ended if at least one out of the three criteria shown in Equation (15) is met, unless the iteration counter reaches a maximum value (e.g., 100):

(15) $$\begin{array}{ccc} \max\left(\left|{\underline{\underline{J}}}^{T}\underline{\underline{W}}\underline{r}\right|\right)<\varepsilon_{1}; & \max\left(\left|\frac{{\underline{h}}_{LM}}{\underline{\Delta H}}\right|\right)<\varepsilon_{2}; & \frac{\chi^{2}}{k-1}<\varepsilon_{3} \end{array}$$

It is also worth highlighting that although all the (dimensionless) user parameters, i.e., *ε*_0_, *ε*_1_, *ε*_2_, *ε*_3_, *λ*_0_, *λ_UN_* and *λ_DN_*, defined earlier in this section are common to any LM implementation, their selection must be done in view of the specific application.

## 3. Experimental Flight Test Campaign

An experimental campaign of flight tests has been carried out to validate and assess the performance of the proposed cooperative strategy for the calibration of magnetic sensors installed on board MAVs. Specifically, the main performance parameter is the accuracy level charactering the estimation of the calibrated magnetic heading obtained applying Equation (6). To evaluate this accuracy, a reference estimate of the heading angle must be derived to be used as a ground truth (as described in detail in the following). This section is divided in two parts corresponding to different experimental tests carried out using, as deputy, a fixed ground station and a flying vehicle, respectively.

### 3.1. Experiment with a Ground-Fixed Deputy

#### 3.1.1. Experimental Setup

**Figure 3:**
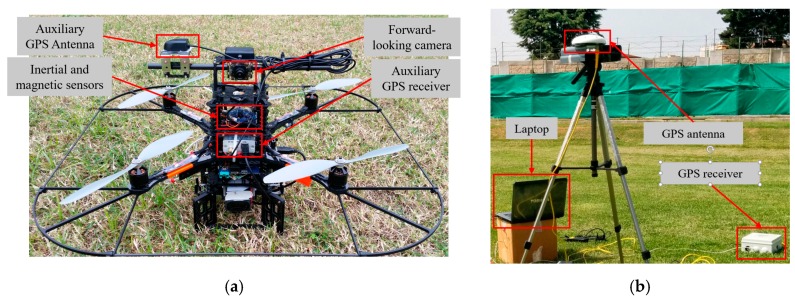
(**a**) Pelican quadcopter used as chief UAV. (**b**) Fixed ground station used as deputy.

For the first experiment, the chief is a customized version of the Pelican^TM^ quadrotor from Ascending Technologies^TM^ (Krailling, GermanY; http://www.asctec.de/) while the role of the deputy is played by a fixed ground station, i.e., a laptop connected via serial RS-232 and a serial-USB adapter to a GNSS antenna (AV59) and receiver (BD960) both produced by Trimble^TM^ (Sunnyvale, CA, USA; https://www.trimble.com/). The chief UAV and the deputy ground station are depicted in Figure 3.

The chief uses a 752 × 480 miniaturized CMOS forward looking camera, i.e., the mvBlueFOX-MLC-200wc produced by Matrix Vision^TM^ (Oppenweiler, Germany; https://www.matrix-vision.com/), to image the deputy. Since the Pelican autopilot does not give access to the GNSS raw data (which are required to enable DGPS or CDGPS processing), an auxiliary antenna and GNSS receiver (LEA 6T) produced by uBlox^TM^ (Thalwil, Switzerland; https://www.u-blox.com/en) are installed on the chief. Inertial and magnetic sensor measurements are collected at a frequency of 40 Hz, while images and GNSS data are gathered simultaneously at a frequency of 1 Hz. To ensure the synchronization of GNSS, visual and inertial/magnetic data, they must be referred to the same time scale. This task is achieved by using an acquisition software [11] developed to save all the sensor data with an accurate time-tag based on the CPU clock. This time-tag is associated with GNSS measurements, which include the GPS time, with very small latency. Moreover, a precise visual/GNSS synchronization is attained since the image acquisition is triggered by the reception of the first GPS package. The deputy ground station also collects GNSS raw data at 1 Hz.

Despite a single deputy suffices for the application of the proposed calibration approach, an additional ground station is positioned on the test field to collect GNSS raw data. Indeed, if both the ground stations are contained in the field of view of the camera on board the chief during the entire flight test, the DGPS-vision algorithm described in [11] can be adopted to obtain the desired reference solution for the heading angle of the chief. This quantity, indicated as *ψ_DGPS-VISION_* in the following, has an average accuracy of the order of 0.5° (which is far better than the heading accuracy provided by the Pelican onboard autopilot) [11].

In this work, the proposed magnetic calibration strategy is carried out using off-line the data collected in flight. Of course, to enable an autonomous real-time implementation, a Vehicle-2-Vehicle (V2V) communication link is required to exchange the navigation data. In this respect, it is worth outlining that the amount of information to be exchanged (i.e., the GNSS data) is limited to a few Kbits per second, thus being compatible with the specifications of standard V2V communication systems [37].

#### 3.1.2. Results

**Figure 4:**
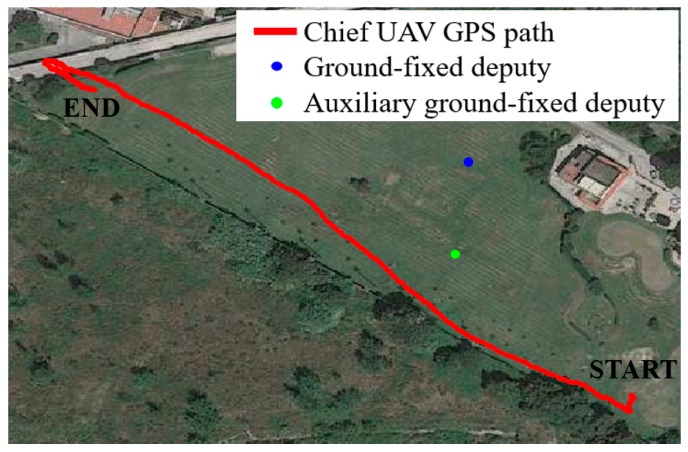
Flight path of the chief (UAV to be calibrated) during the experimental test. The locations of the deputies (ground stations) on the test site are also highlighted.

The magnetic calibration strategy is applied over data collected during a single flight test, lasting around 8 minutes (although this relatively long time duration is not strictly required for the applicability of the method). The flight path travelled by the chief on the testing site is depicted in Figure 4, where the positions of the two ground stations are also indicated. Among them, the farthest one with respect to the chief (blue dot in Figure 4) is selected as deputy for the cooperative calibration process to limit the uncertainty in the GNSS-based estimation of *u_NED_*. The other ground station (green dot in Figure 4) is indicated as auxiliary deputy as it is used to allow the estimation of the reference heading solution (*ψ_DGPS-VISION_*) according to the DGPS-vision algorithm in [11].

**Figure 5:**
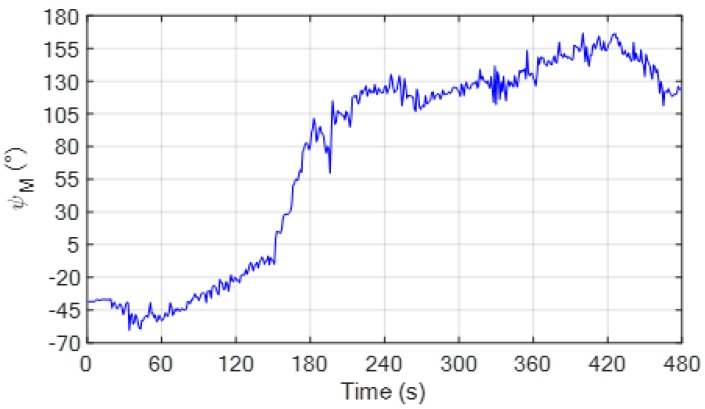
Time variation of the non-calibrated magnetic heading (*ψ_M_*), obtained applying (4), during the flight test with a fixed deputy. The local magnetic declination at the test site and date of the experiment is 3.07°.

The flight path is assigned to provide a significant variation of the heading of the chief. This aspect is important to ensure an adequate sampling of the residual vector function for the minimization process. The time variation of the non-calibrated magnetic heading, obtained applying (4), is depicted in Figure 5. For this computation, the local magnetic declination is obtained using an on-line free software [38], which requires in input the location of the test site (latitude, longitude and altitude) and the time of the experiment. In this case, *δ_M_* is 3.07°.

**Figure 6:**
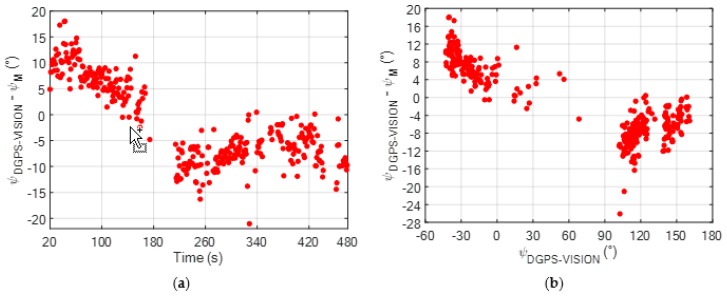
(**a**) Time variation of *ψ_ERR_* for the non-calibrated magnetic heading (*ψ_M_*) during the flight test; (**b**) Variation of *ψ_ERR_* for the non-calibrated magnetic heading (*ψ_M_*) as a function of the reference heading solution obtained using the DGPS-vision algorithm [11].

To highlight the effect caused by the presence of onboard magnetic disturbances on the magnetic heading estimation, the heading error (*ψ_ERR_*), measured as the difference between *ψ_DGPS-VISION_* and *ψ_M_*, is depicted in Figure 6. Specifically, *ψ_ERR_* is expressed as a function of time and of the reference *ψ_DGPS-VISION_* estimate in Figure 6a,b, respectively.

The starting time in Figure 6a is 20 s. In fact, the time interval during the power on of the electric engines and the takeoff can be neglected as it does not correspond to a nominal flight condition (and, consequently, nominal value of the magnetic bias caused by onboard sources). It is worth outlining that the presence of gaps in the time variation of *ψ_ERR_* in Figure 6a is due to temporary absence of the reference heading solution (*ψ_DGPS-VISION_*), mainly occurring if the ground stations are not both enclosed in the camera FOV. By looking at both Figure 6a,b, it is clear how the time variation of *ψ_ERR_* is correlated to the variation of the true heading. This is due to the magnetic bias vector (*∆H*) being constant in BRF. Specifically, the sign of the magnetic heading estimation error is positive for negative heading, while it becomes negative as the chief is pointed with a positive heading.

**Figure 7:**
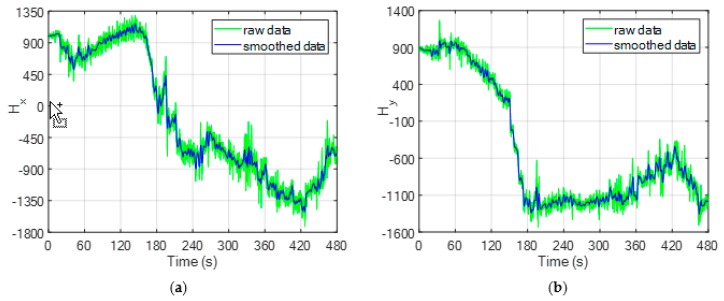
Time variation of the magnetometer measurements during the flight test. The raw data (green line) are smoothed using a moving average operator obtaining a lower-noise dataset (blue line). (**a**) Component of *H* along the *x* axis of BRF; (**b**) Component of *H* along the *y* axis of BRF. Please note that these magnetometer measurements, provided by the autopilot installed on board the chief by Ascending Technologies^TM^, are dimensionless quantities. In this respect, consider that the norm of the Earth magnetic field vector at date and location of the flight test is 46,126 nT.

Both for the computation of *ψ_M_* as shown in Figure 5, and for the calibration procedure, the raw magnetic sensor measurements must be filtered to cope with the associated high level of noise. Here, this is done by applying a moving average operator to these noisy magnetic data. A smoothing time of 2.5 s is set when applying the moving average operator considering the relatively smooth flight dynamic and the low magnetometer bandwidth. The result of this operation for the components of *H* along the *x* and *y* axes of BRF is shown by Figure 7.

At this point, the LM-based calibration algorithm can be run. To this end, a key role is played by the selection of the *k* time instants at which the visual and GNSS data are used. This is done by considering two main guidelines:

- Time instants at which the yaw rate, evaluated considering the gyro measurement along the *z* axis of BRF, is larger than a threshold (*τ_ω,z_*) should be excluded.
- Time instants at which the chief-deputy relative distance is lower than a threshold (*τ_R_*) should be excluded.

The first condition is set to minimize the heading variation associated to the selected time instants considering the low bandwidth of the magnetometer. The second condition allows limiting the DGPS uncertainty which characterizes the estimation of *u_NED_*. In this work, *τ_ω,z_* is set to 0.25°/s, while *τ_R_* is set to 70 m, thus allowing the selection of *k* = 18 time instants. The heading value at the selected frames varies from −38.5° to 125.3°, while the mean chief-deputy relative distance is 105.12 m. With regards to the selection of the user’s parameters for the LM implementation (defined in Section 2.3), the adopted setting is reported in Table 1.

The initialization of the damping factor is chosen to favor the Gauss-Newton minimization process with respect to the gradient descent approach, while the values set for *λ_UN_* and *λ_DN_* have shown to provide good convergence in the LM optimization process [30]. With regards to the convergence thresholds, *ε*_2_ is the largest (10^−4^) since it depends on the desired accuracy in the estimated value of Δ*Hx_S_* and Δ*Hy_S_*. In this respect, a variation of Δ*Hx_S_* and Δ*Hy_S_* in the order of *ε*_2_ must have a negligible effect on the value of *r*. Given this parameters’ setting, the convergence is reached simultaneously for both the first (1 × 10^−11^) and second (8 × 10^−5^) condition in Equation (15) after five iterations, while the value of the third convergence metric, i.e., *χ*^2^/(*k*−1), stops at (5 × 10^−4^). The resulting values of Δ*Hx_S_* and Δ*Hy_S_* are −267.53 and −4.17, respectively.

**Figure 8:**
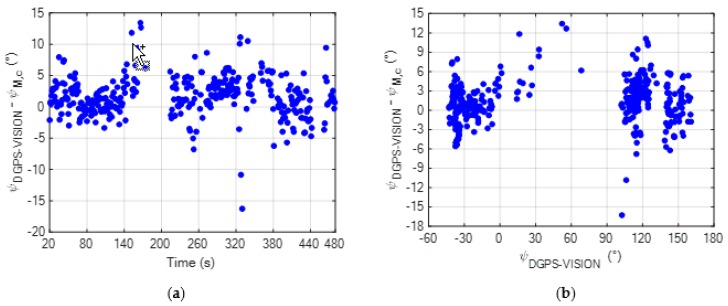
(**a**) Time variation of *ψ_ERR_* for the calibrated magnetic heading (*ψ_M,c_*) during the flight test; (**b**) Variation of *ψ_ERR_* for the calibrated magnetic heading (*ψ_M,c_*) as a function of the reference heading solution obtained using the DGPS-vision algorithm [11].

Using these components of the magnetic bias vector in BSRF, the calibrated magnetic heading can be computed using (6), and the resulting estimation error is reported in Figure 8, again as a function of time and *ψ_DGPS-VISION_*.

By looking at Figure 8b, it can be stated that the proposed calibration approach allows removing the bias induced in the magnetic heading estimation by onboard sources, as the resulting error is independent of *ψ_DGPS-VISION_* (unlike in Figure 6b for the non-calibrated magnetic heading). A statistical comparison between the accuracies characterizing *ψ_M_* and *ψ_M,c_* is reported in Table 2, considering the absolute value of *ψ_ERR_* as a performance metric.

The statistics reported in the table above quantitatively show the achievable improvement in the magnetic heading accuracy. Specifically, the absolute value of the bias reduces by about 5°, while the standards deviation reduces by about 1°.

### 3.2. Experiment with a Flying Deputy

#### 3.2.1. Experimental Setup

Additional flight tests are conducted using a flying vehicle as deputy for the magnetic calibration process. The advantages of using another UAV as deputy, instead of a fixed ground station, are mainly related to the possibility to realize more quickly the data collection process (while meeting all the identified guidelines regarding the true heading, the range and the yaw rate), especially if the pre-defined coordinated maneuver is executed autonomously. Moreover, as already mentioned, if each UAV is able to visually detect and track the other, the collected data can be used for the calibration of both their corresponding magnetic sensors.

**Figure 9:**
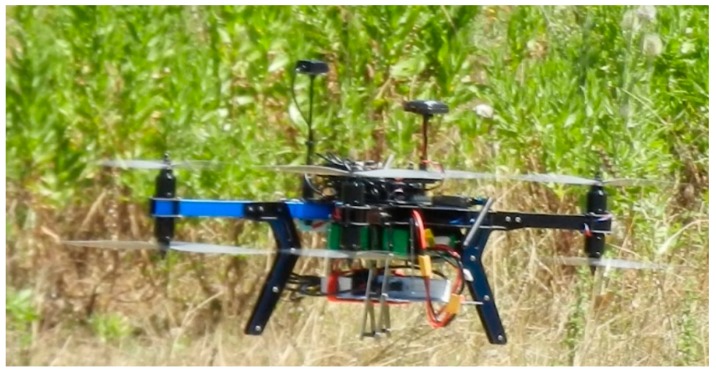
Customized X8+ by 3D Robotics used as flying deputy.

The chief UAV is the same described in Section 3.1.1. Instead, the role of the deputy is played by a X8+ octocopter produced by 3D Robotics^TM^ (Berkeley, CA, USA; https://3dr.com/) depicted in Figure 9. Just like the chief, the deputy is customized by the installation of an auxiliary GNSS receiver and antenna to enable the acquisition of the GNSS raw data. The GNSS receiver is connected via USB to an embedded CPU, i.e., the Odroid XU4^TM^ now produced by Hardkernel (Anyang, South Korea; https://www.hardkernel.com/), for data acquisition and storage.

Besides the deputy, the same ground stations as described in Section 3.1.1 are also installed on the test field and they are always kept in the field of view of the camera to allow the estimation of the reference heading solution. Finally, the same acquisition software and data synchronization strategy described in Section 3.1.1, have been used for these flight experiments. The only difference lies in the fact that inertial data are collected at a higher data rate (i.e., around 76 Hz).

#### 3.2.2. Results

Three flights are carried out at a different test site, so the local value of *δ_M_* at the time of the experiments is 3.21°. The goal of these additional tests is twofold. On one side, the collected data can be used to further validate the reliability of the proposed method by applying the values of Δ*Hx_S_* and Δ*Hy_S_* computed in Section 3.1.2 to correct the bias affecting the estimated magnetic heading. On the other side, the LM-based calibration algorithm can be applied again to check the consistency of the method.

**Figure 10:**
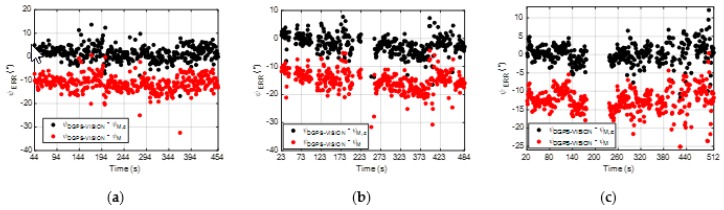
Time variation of *ψ_ERR_* for the calibrated (*ψ_M,c_*) and non-calibrated (*ψ_M_*) magnetic heading during the three flight tests conducted with a flying deputy. *ψ_M,c_* is obtained applying (6) with the values of the magnetic bias vector components obtained from the calibration results described in Section 3.1.2, i.e., *ΔHx_S_* = −267.53, *ΔHy_S_* = −4.17. (**a**) Flight test 1; (**b**) Flight test 2; (**c**) Flight test 3.

With regards to the first analysis, the time variation of *ψ_ERR_* during the three flight tests for both the calibrated and non-calibrated magnetic heading is reported in Figure 10. Please note that for all the three cases, the time intervals during which the engines are not operating nominally, e.g., before takeoff and after landing, are not considered. Also, during the second and third flight, a set of 360° turns is commanded to the chief, so in the corresponding time interval the ground stations disappear from the FOV and the reference *ψ_DGPS-VISION_* cannot be computed.

First, it is interesting to note that, since the true heading is positive during the three flight tests, the bias in *ψ_M_* is always negative. This is in line with the dependence of the magnetic heading error on the true heading observed in Figure 6b. Overall, Figure 10 shows that the magnetic bias vector components derived thanks to the calibration process described in Section 3.1.2 can be used to correct the magnetometers measurements relative to these new flight tests, thus significantly reducing the systematic error in *ψ_M,c_* with respect to *ψ_M_*. To synthetize these results, a statistical comparison between the accuracies characterizing *ψ_M_* and *ψ_M,c_* is reported in Table 3, considering the absolute value of *ψ_ERR_* as the performance metric.

By looking at these statistics, first, it is important to highlight that the noise in the non-calibrated magnetic heading (measured by the standard deviation of ***|ψ_ERR_|***) has the same level for all the flight tests analyzed in this work. Instead, the bias (measured by the mean of ***|ψ_ERR_|***) is larger in the latter three flight tests (i.e., it varies from 11.5° to 15.2°) than in the one analyzed in Section 3.1.2 (i.e., 7.4°). This occurs since the constancy of ∆*H* in BRF induces a bias in the magnetic heading which is a function of the true heading (as highlighted, for instance, in Figure 6b). In the case of the three flights analyzed in this section, the average pointing of the chief (determined by the main direction of the test field with respect to the geographic North) provides a less favorable condition for the vector composition of *H_E_* and ∆*H* (considering that ∆*H* has its main component along the *x* axis of BSRF). However, by applying the correction on the magnetic sensor measurements computed by the proposed calibration approach, both the bias and the noise in the estimated magnetic heading are significantly reduced.

Finally, the proposed calibration approach is applied again, but using the data collected during one of the flight tests performed with a flying deputy, in order to show the consistency of the method. In this respect, the third flight is selected as it is characterized by the larger variability in the true heading (which allows better sampling the residual vector function). As *τ_ω,z_* and *τ_R_* are again set to 0.25°/s, and 70 m, respectively, the visual and GNSS data required for the calibration are relative to *k* = 14 time instants. The heading value at the selected frames varies from 47.4° to 87.2°, while the mean chief-deputy relative distance is 107.71 m.

By keeping the same LM parameters listed in Table 1, the resulting values of Δ*Hx_S_* and Δ*Hy_S_* are −275.53° and 41.16°, respectively. A statistical comparison between this solution and the one obtained in Section 3.1.2 is carried out in terms of ***|ψ_ERR_|*** computing the calibrated magnetic heading for the third flight test. The results, reported in Table 4, confirm that the same level of accuracy is kept, despite a slight change in the component of ∆*H* along the *y* axis of BSRF (which is still much lower than Δ*Hx_S_*).

## 4. Conclusions

An innovative method for the calibration of low-cost magnetometers for small/micro unmanned aerial vehicles was presented. It aims at improving the accuracy in the magnetic heading estimate by correcting the systematic error caused by the presence of onboard magnetic fields during nominal flight conditions (e.g., generated by the engines and, potentially, by other electric devices). This problem was addressed by collecting calibration data in flight and adopting a cooperative approach. In fact, the proposed method requires the unmanned aerial vehicle to be calibrated (chief) to visually detect and track a cooperative deputy, both placed under nominal Global Navigation Satellite System coverage to allow relative positioning. If the deputy is a flying vehicle a coordinate flight maneuver must be executed. Using the collected data, the calibration problem can be formulated as a system of non-linear equations solved applying the Levenberg-Marquardt algorithm. An experimental campaign of flight test was conducted to assess the feasibility of the proposed approach and to quantitatively evaluate the achievable magnetic heading accuracy, using either a fixed ground station or a flying vehicle as deputy. Results showed that onboard magnetic sources, being constant in the body reference frame of the chief, induce a systematic error in the estimated magnetic heading, whose entity and sign are a function of the true heading of the chief. This effect is removed by applying the magnetic bias vector components estimated by the calibration procedure to correct the magnetometers measurement. In fact, the residual error in the calibrated magnetic heading is reduced down to two degrees in all the analyzed tests, and it is independent of the true heading of the chief. Besides the systematic error, the noise in the estimated magnetic heading is also slightly reduced after the calibration.

Future research activities will be dedicated to analytically deriving the sensitivity of the calibration approach with respect to the uncertainty in the calibration data (both visual and relative positioning ones). This can also lead to the definition of a standard procedure for data collection which can be efficiently executed exploiting fully autonomous flight mode of the chief and deputy unmanned aerial vehicles. Finally, additional efforts can be addressed to develop a generalized calibration approach able to account also for magnetic disturbances caused by external sources.

## Figures and Tables

**Table 1:** Dimensionless parameters characterizing the LM-based magnetic calibration algorithm.

*ε*_0_	*ε*_1_	*ε*_2_	*ε*_3_	*λ*_0_	*λ_UN_*	*λ_DN_*
10^−4^	10^−10^	10^−4^	10^−6^	10^−5^	11	9

**Table 2:** Time statistics of the absolute value of the magnetic heading estimation error. Calibrated vs. non-calibrated case. The symbol |∙| indicates the absolute value operator.

Magnetic Heading	*|ψ_ERR_|* (°)	
	Mean	Standard Deviation
*ψ_M_*	7.4	3.6
*ψ_M,c_*	2.7	2.4

**Table 3:** Time statistics of the absolute value of the magnetic heading estimation error for the three tests with a flying deputy. Calibrated vs. non-calibrated case. The symbol |∙| indicates the absolute value operator. The magnetic heading is corrected using the magnetic bias vector components in BSRF as computed in Section 3.1.2 (Δ*Hx_S_* = −267.53, Δ*Hy_S_* = −4.17).

Magnetic Heading (Flight Test)	*|ψ_ERR_|* (°)	
	Mean	Standard Deviation
*ψ_M_* (1)	11.5	3.4
*ψ_M,c_* (1)	2.5	2.2
*ψ_M_* (2)	15.2	3.6
*ψ_M,c_* (2)	3.2	2.5
*ψ_M_* (3)	12.5	3.2
*ψ_M,c_* (3)	2.1	1.9

**Table 4:** Time statistics of the absolute value of the estimation error on the calibrated magnetic heading for the third flight test with a flying deputy. Comparison between two solutions for the magnetic bias vector components.

Magnetic Bias Vector Components in BSRF	*|ψ_ERR_|* (°)	
	Mean	Standard Deviation
Flight test with a fixed deputy (Section 3.1.2) Δ*Hx_S_* = −267.53, Δ*Hy_S_* = −4.17	2.08	1.94
Flight test 3 with a flying deputy Δ*Hx_S_* = −275.53, Δ*Hy_S_* = −41.16	2.02	1.94
